# Antibiotics and Missed Etiological Diagnosis of Infective Endocarditis: A Dangerous Duo

**DOI:** 10.3390/jcm11154533

**Published:** 2022-08-03

**Authors:** Daniele Roberto Giacobbe, Antonio Salsano, Francesco Santini, Matteo Bassetti

**Affiliations:** 1 Department of Health Sciences (DISSAL), University of Genoa, Via A. Pastore 1, 16132 Genoa, Italy; matteo.bassetti@unige.it; 2 Infectious Diseases Unit, IRCCS Ospedale Policlinico San Martino, 16132 Genoa, Italy; 3 Department of Integrated Surgical and Diagnostic Sciences (DISC), University of Genoa, 16132 Genoa, Italy; antonio.salsano@unige.it (A.S.); francesco.santini@unige.it (F.S.); 4 Division of Cardiac Surgery, IRCCS Ospedale Policlinico San Martino, 16132 Genoa, Italy

The etiological diagnosis of infective endocarditis (IE) still remains a challenge. Aside from IE caused by nutritionally variant streptococci, intracellular bacteria, and microorganism belonging to the HACEK group, all of which are possible causes of culture-negative IE (CNIE), the most frequent cause of CNIE is likely the unnecessary administration of antibiotics before blood cultures collection in patients with stable clinical conditions and not requiring immediate medical therapy [1,2,3,4]. The unfavorable impact of such practice may be subtle and not immediately perceived at the bedside of patients, since the final outcome of IE could manifest much later than the initial diagnostic assessment, when the consequences of initial treatment decisions are less evident. However, over the years, several studies have suggested a less favorable prognosis of CNIE compared with culture-positive IE (CPIE) and, in our opinion, this accumulating evidence should no longer be ignored.

As far back as 2001, Zamorano and colleagues reported on the prognosis of 103 consecutive patients with IE, of whom 20 (19%) and 83 (81%) had CNIE and CPIE, respectively [5]. While mortality was similar between patients with CNIE and those with CPIE (15% and 13%, respectively), patients with CNIE had a higher frequency of complications than patients with CPIE in terms of heart failure (50% vs. 20%) and valve rupture and/or perforation (40% vs. 10%) [5]. A few years later, Murashita and colleagues reported on the outcome of 67 patients who underwent prosthetic valve replacement for IE, of whom 21 had CNIE (31%) [6]. While CNIE was not associated with early mortality (although the direction of the effect was nonetheless toward unfavorable prognosis, with an odds ratio [OR] of 4.57 and a 95% confidence interval [CI] from 0.91 to 22.8, *p* = 0.064), CNIE showed an association with late mortality (OR 3.14 with 95% CI from 1.1 to 9.1, *p* = 0.0354) [6]. In a large, prospective cohort of 2000 patients with IE, of whom 290 had CNIE (15%), the absence of etiological diagnosis was independently associated with increased in-hospital mortality in multivariable analysis (adjusted OR 1.8 with 95% CI from 1.1 to 2.9, *p* = 0.016) [7]. In a recent analysis of the large ESC-EORP EURO-ENDO registry, out of 3113 with IE, 523 patients had CNIE (17%). Overall, CNIE was independently associated with 1-year mortality (hazard ratio [HR] 1.3 with 95% CI from 1.0 to 1.6, *p* = 0.02), but this association was not appreciable in the subgroup of patients who underwent surgery [8]. Notably, in the ESC-EORP EURO-ENDO registry congestive heart failure was more frequent in CNIE than in CPIE patients, as also previously observed by other authors [3,5]. It has been suggested that this could be related to a delayed recognition of IE in the patients with negative blood cultures, with their severe conditions at diagnosis contraindicating surgery and negatively impacting prognosis [8]. From this standpoint, variations in the frequency of CNIE patients in whom surgery was contraindicated, together with possible differences in the appropriateness (unverifiable due to the lack of etiological diagnosis) of the antibiotic therapy of CNIE, could explain why a few other studies did not register a worse prognosis in CNIE than in CPIE [9,10,11].

In our opinion, all the studies discussed above may support the impression that combining proper antibiotic use with prompt recognition of IE before severe complications develop are pivotal in reducing CNIE rates and mortality. Certainly, in the near future molecular diagnostics will also contribute (in part they already do) to further reduce the absolute frequency of CNIE. However, this does not mean that we can forget to use antibiotics wisely, since only a proper synergy between novel diagnostics and judicious antibiotic use may eventually allow us to maximize etiological diagnosis. Finally, it should be reminded that achieving the etiological diagnosis of IE also offers other crucial advantages (e.g., narrow-spectrum antibiotic therapy, possible step-down to oral therapy). This is a target we should no longer miss.
